# Comparison of Invasive Ductolobular Carcinoma and Lobular Carcinoma: An Observational Study

**DOI:** 10.3390/medicina61020310

**Published:** 2025-02-10

**Authors:** Mahmut Uçar, Mukaddes Yılmaz, Eda Erdiş, Birsen Yücel

**Affiliations:** 1 Department of Medical Oncology, Sivas Cumhuriyet University, 58140 Sivas, Turkey; ylmzmukaddes@gmail.com; 2 Department of Radiation Oncology, Sivas Cumhuriyet University, 58140 Sivas, Turkey; dr.erdiseda@gmail.com (E.E.); yucelbirsen@yahoo.com (B.Y.)

**Keywords:** breast cancer, mixed ductolobular carcinoma, invasive lobular carcinoma

## Abstract

*Background and Objectives:* Mixed ductolobular carcinomas (mDLCs) are tumors that contain both ductal and lobular components. The clinicopathological characteristics and impacts on survival of the two components, which have distinct biological behaviors, are still not clearly understood. This study aimed to compare the clinicopathological characteristics, recurrence/metastasis patterns, and survival outcomes of mDLC and invasive lobular carcinoma (ILC), as well as to investigate the prognostic significance of both histopathologies. *Materials and Methods*: The outcomes of 132 patients who were followed and treated between 2010 and 2021 were analyzed. Patients were examined in two groups, ILC and mDLC. Chi-square tests were performed to compare the baseline clinicopathological characteristics and treatments. Survival rates were subsequently analyzed using the Kaplan–Meier method and compared using the Cox proportional hazards model. *Results:* In this study, 80 (61%) patients had ILC histopathology, while 52 (39%) had mDLC histopathology. Differences between the groups were observed in median age (*p* = 0.038), N stage (*p* = 0.046), estrogen receptor (ER) status (*p* = 0.005), lymphovascular invasion (*p* = 0.007), median tumor diameter (*p* = 0.050), and frequency of distant metastasis (*p* = 0.029). The treatments, relapse patterns, and metastasis patterns were similar (*p* > 0.05). No differences in overall survival (OS) and disease-free survival (DFS) were observed. In the multivariate analysis, mDLC histopathology was identified as a poor prognostic factor (HR: 2.95, CI 95%: 1.10–7.88, *p* = 0.030). Histopathology (ILC vs. mDCL) was not identified as a prognostic factor in the Cox regression analysis for DFS. *Conclusion:* Although mDLC has poor clinicopathological features (younger age, more advanced N stage, more ER negativity, more lymphovascular invasion, and more frequency of metastases) and appears more aggressive than ILC, these changes do not affect survival in this study. However, mDLC histopathology seems to be associated with poor prognosis for OS.

## 1. Introduction

The architectural structure of tumor cells, as well as their morphological and immunostaining characteristics, have resulted in the identification of histopathological subgroups that alter the biological behavior and clinical course of breast cancer [1]. Invasive ductal carcinoma is the most common histological type of breast cancer, constituting 40–80% of all breast cancers [2]. However, this group also exhibits some morphological differences. When these differences are sufficient and distinct, they are termed specific types of invasive ductal carcinoma (IDC); when they are not distinct, they are referred to as invasive ductal carcinoma that is not otherwise specified (NOS). Invasive lobular carcinoma (ILC), following IDC in terms of incidence, constitutes 5–15% of all breast cancers and is a morphological type associated with the loss of the cell adhesion molecule E-cadherin, which causes cancer development [3,4]. ILC is typically seen in older patients, expresses strong estrogen and progesterone receptors, often lacks HER-2 expression, and tends to be multicentric and of a low grade [3,5].

ILC can coexist with other types of invasive carcinoma, most commonly with IDC [4,6]. In such cases, terms such as mixed lobular–ductal carcinoma, lobular-featured invasive ductal carcinoma, and invasive mixed ductolobular carcinoma (mDLC) are used [4]. mDLC constitutes approximately 5% of all breast cancers [6]. These tumors exhibit morphological heterogeneity, which is probably due to the different mutations present in the tumor at the molecular level. Normal membranous E-cadherin expression is observed in ductal foci, while lobular foci may show complete loss of staining or abnormal and/or positive staining. This suggests that during tumor development, the loss of E-cadherin from a ductal focus can lead to a lobular phenotype [7].

These mixed tumors, containing clonal and morphological changes, can exhibit different clinical and pathological characteristics from those of ILC. When genetically analyzed, mDLC has been defined as two groups with different genetic characteristics, which are termed ILC-like and IDC-like. The behavior of mDLC is thought to be determined by its genetic characteristics [8]. The outcome of mixed tumors is influenced by the presence of poor prognostic components. However, accompanying components with a better prognosis may not be decisive in the course of the disease [9]. In their study of patients with stage I–III breast cancer, by searching the National Cancer Database, Lohami et al. showed that mDLC has the clinicopathological characteristics of both IDC and ILC [10]. However, the features of mDLC were found to be intermediate between those of IDC and ILC, and mDLC behaves more like ILC. In a study by Rakha et al., mixed tumors were found to occur in younger premenopausal patients, to have higher histological grades, to exhibit expression of P-cadherin, and to lack androgen, BRCA1, and FHIT proteins, which are poor prognostic markers. Local–regional recurrence and metastasis were more frequent, and survival was slightly worse compared with ILC, which was in line with the negative prognostic indicators. Moreover, different histologies in different organ metastases within the same patient have been demonstrated [11]. In their study, Zengel et al. stated that mDLC has different histopathological characteristics, more commonly occurs in older patients, and shows no difference from ILC in terms of survival [12]. In another study, the clinicopathological characteristics of mDLC were compared with those of IDC and ILC, and mDLC was found to exhibit features that were more compatible with ILC [13]. Otto et al. demonstrated that mDLC had a better prognosis than ILC, especially in postmenopausal patients, that histological grade could be used as a prognostic marker, and that aromatase inhibitors had a favorable efficacy compared with tamoxifen in adjuvant hormone therapy [14]. Zels et al. showed that biopsies from metastases in patients with metastatic mDLC have lobular features [15]. The pathological features and clinical significance of mixed cancer, which is formed by a more aggressive subtype added to a specific type, are of great interest. Their rarity makes it difficult to organize clinical trials with sufficient sample sizes and timely accrual of subjects. Treatment algorithms for mixed tumors are limited.

In this retrospective study, the clinicopathological features, recurrence/metastasis patterns, and survival outcomes of mDLC and ILC were compared, and the prognostic significance of both histopathologies was investigated.

## 2. Materials and Methods

### 2.1. Study Design

This study was planned as a comparative retrospective observational study. Approval from the local ethics committee was obtained prior to commencement, and the ethical principles outlined in the Declaration of Helsinki were adhered to. Due to the retrospective design and anonymity of the study, written consent was not obtained from the patients.

### 2.2. Population and Sample

In this study, 1611 breast cancer patients who were followed and treated at Sivas Cumhuriyet University Faculty of Medicine Oncology Center between 2010 and 2021 were screened. A total of 52 (3%) patients with histological mDLC and 80 (5%) patients with pure ILC were included in the study. Patients over the age of 18 at stages I–IV were included. Patients with other histological types, those missing follow-up information, those who dropped out of follow-up, and those with secondary malignancies were excluded from the study. To avoid sampling bias, self-selection bias, and survivorship bias in the target population selected, all patients admitted within a certain date range were included in the study, and the last patient enrolled in the study was selected to provide survival data for at least 3 years. To avoid undercoverage bias, the inclusion and exclusion criteria were met.

### 2.3. Data Collection

Demographic data such as age, menopausal status, Eastern Cooperative Oncology Group (ECOG) performance status, and tumor characteristics, including histological type, estrogen receptor (ER), progesterone receptor (PR), human epidermal growth factor receptor 2 (HER2) status, grade, Ki-67 score, location, metastatic status, and organs involved, as well as treatment details, were obtained from the hospital’s medical record system.

The diagnosis and staging of breast cancer (BC) were performed according to the seventh edition of the AJCC guidelines [16]. The final diagnoses were made according to the international histological classification from the World Health Organization (WHO) [6]. The histological grades were determined using the modified Scarff–Bloom–Richardson grading system [17]. Positive nuclear immunohistochemical staining of at least 1% of the tumor cells was considered to indicate ER and PR positivity [1]. On the other hand, weak to moderate (2+) or strong (3+) results or immunohistochemical staining, along with fluorescence in situ hybridization (FISH) findings, were considered to indicate HER2 positivity.

The intrinsic BC subtypes were determined based on the status of ER, PR, HER2, and Ki-67. BC patients with ER and PR positivity and HER2 negativity were considered to have the luminal-A subtype, whereas BC patients with ER or PR positivity and HER2 negativity were considered to have the luminal-B HER2-negative subtype. In addition, patients with ER and/or PR positivity and HER2 positivity were considered to have the luminal-B HER2-positive subtype. The HER2-positive subtype included only HER2-positive cases. Patients with ER, PR, and HER2 negativity were considered to have the triple-negative subtype [18].

The period from the date of diagnosis to the last follow-up or death was assessed as overall survival (OS), and the period from the date of diagnosis to the date of recurrence/distant metastasis, date of death, or, for those without recurrence/metastasis, the last follow-up date was assessed as disease-free survival (DFS).

### 2.4. Statistical Analysis

SPSS Version 23 (IBM Corp., Armonk, NY, USA) was used for statistical analysis. For non-categorical variables such as age and the values of Ki-67, CEA, and CA 15,3, Student’s *T*-test (normal distribution) or the Mann–Whitney U test (abnormal distribution) were used to compare the groups. The Chi-square test was used to compare categorical variables (menopausal status, presence of comorbidities, performance status, ER and PR status, stage, etc.). The Kaplan–Meier test was used to determine survival times. Factors affecting survival were analyzed via univariate analysis using the long-rank test. The possible factors identified with univariate analyses were further entered into the Cox regression analysis with backward selection to determine independent predictors of survival. If there were missing data, pairwise deletion was applied. A *p*-value of <0.050 was considered statistically significant.

## 3. Results

mDLC patients were observed to be younger, have a more advanced N stage, have more ER negativity, and have more lymphovascular invasion (LVI), but they had a relatively smaller tumor diameter. No differences were observed between the groups in terms of menopausal status, T stage, histological subtypes, PR status, HER2 status, grade, other histopathological features (perineural invasion (PNI), the intraductal component, multicentricity/multifocality, tumor necrosis, extracapsular invasion (ECE)), or Ki-67 values. The clinicopathological characteristics of the patients are provided in Table 1.

In both groups, while neoadjuvant treatment was less commonly used, the majority of patients received adjuvant chemotherapy, adjuvant radiotherapy, and hormone therapy following modified radical mastectomy. The treatments of the patients are summarized in Table 2.

The median follow-up duration for patients was determined to be 85 months (range: 3–252 months). While local recurrence rates were similar across groups, the frequency of metastasis development was significantly higher in the mixed group (*p* = 0.029). While solid organ metastasis was similar in both groups, bone metastasis was numerically higher in the mixed group, though it remained statistically insignificant at the borderline. No differences were detected in either overall survival (OS) or disease-free survival (DFS) between the two patient groups. The comparison of recurrence/metastasis patterns and survival outcomes is presented in Table 3.

**Figure 1:**
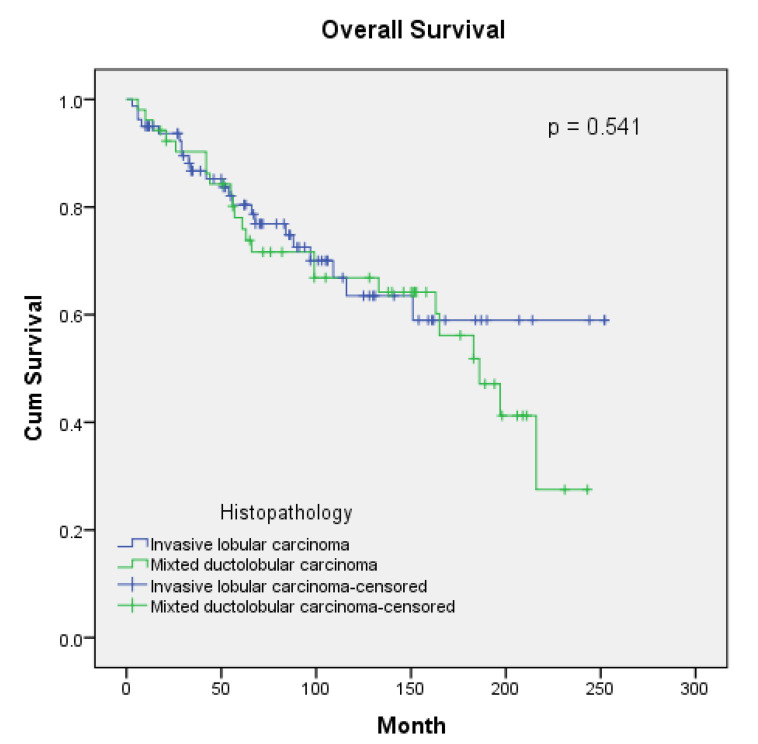
Overall survival curves according to histopathology.

**Figure 2:**
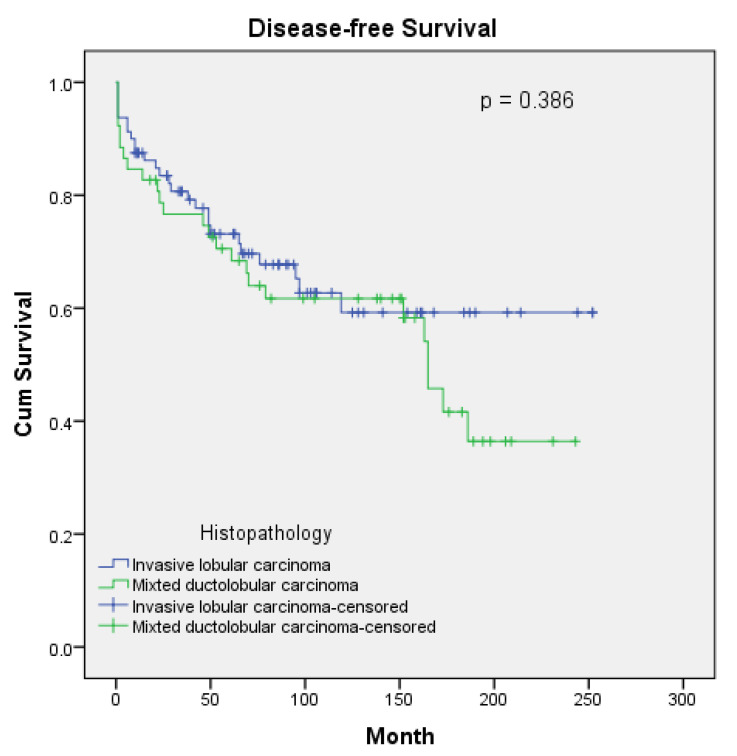
Disease-free survival curves according to histopathology.

The curves for overall survival and disease-free survival are, respectively, shown in Figure 1 and Figure 2. The 5-year, 10-year, 15-year, and median OS for ILC vs. mDLC was 80% vs. 76%, 64% vs. 67%, 59% vs. 52%, and NR vs. 186 months. The 5-year, 10-year, 15-year, and median DFS for ILC vs. mDLC was 73% vs. 68%, 59% vs. 62%, 59% vs. 42%, and NR vs. 165 months (*p* = 0.386).

In the univariate analysis, histopathology was not statistically significant as a prognostic factor affecting OS, but in the multivariate analysis, mDLC was identified as an independent and poor prognostic factor for survival (HR: 2.95, CI 95%: 1.10–7.88, *p* = 0.030). Factors such as menopausal status, histological subtype, T and N stages, grade, perineural invasion (PNI), tumor necrosis, and extracapsular extension (ECE) were identified as prognostic factors affecting OS in univariate analysis, while histopathology, PNI, ECE, and N stage were determined to be independent prognostic factors. The prognostic factors affecting OS in both the univariate and multivariate analyses are shown in Table 4.

For DFS, the T and N stages, grade, PNI, tumor necrosis, and ECE were identified as prognostic factors in the univariate analysis. In the multivariate analysis, ECE was determined to be an independent prognostic factor. The histopathologies that were compared were not shown to be prognostic factors affecting DFS. The prognostic factors affecting DFS in both the univariate and multivariate analyses are presented in Table 5.

## 4. Discussion

In this study, mDLC was observed in 3% of all breast cancer patients, while ILC was observed in 5%. When the clinical and pathological characteristics of the patient groups included in this study were compared, it was observed that mDLC exhibited relatively worse characteristics (younger age, more advanced N stage, higher rate of ER negativity, more presence of LVI, and more distant metastasis frequency) than those of ILC. Despite the worse characteristics in mDLC, no differences in survival for either OS or DFS were detected between the two groups. However, mDLC histopathology was identified as an independent and poor prognostic factor for OS in multivariate analysis.

The incidence of mixed breast cancer is between 3 and 5%, and recent years have shown an increase in the incidence of both ILC and mDLC [7], which is claimed to be due in part to the use of hormone replacement therapy among women [19]. The rarity of such cases makes it difficult to organize clinical trials with sufficient sample sizes. Genetic and geographic factors also vary in terms of the incidence of lobular cancer, which is typically between 5 and 15% [20]. In our study, mDLC and ILC were observed at rates of 3% and 5%, respectively, similarly to the findings in the literature.

Comparing ILC and mDLC, a study by Acs et al. found a lower proportion of patients under 50 years and a lower postmenopausal patient rate in the mixed group [21]. Conversely, a study by Zengel et al. found that while the ages of ILC patients were older, the postmenopausal patient rates were similar [12]. In a series by Duraker et al., patients in the ILC group were older, while the postmenopausal patient rate was also higher [22]. In a series by Metzger-Filho et al., the ILC group consisted of older patients and had a similar proportion of postmenopausal patients [14]. Similarly, comparisons showing ILC occurring at older ages in comparison with mixed cases have been reported in the literature [23]. Consistely with the literature, our study observed that patients with mDLC were younger, although their menopausal status was similarly distributed.

There is a consensus in the literature regarding ER, PR expression, and HER-2 negativity in ILC and mixed diseases [12]. In a study by Nasrazadani et al., the ER positivity rate was found to be 96% for ILC and 91% for mDLC, and a significant difference was identified. HER-2 positivity was low in both groups and was similarly observed. A meta-analysis conducted in the same study that included 23 studies found that hormone receptor positivity was similar between ILC and mDLC [13]. Another study reported that mDLC had lower ER positivity, more frequent progesterone receptor positivity, and more frequent HER-2 positivity [12]. Many studies comparing ILC and mDLC have observed similar hormone receptor positivity across groups [21,24,25]. Similarly, in our study, ER positivity was lower in mDLC, but PR and HER-2 positivity was similar across groups.

The tumor grade is a prognostic marker in breast cancer [26]. ILC and mDLC are generally low-grade tumors [21]. Comparative studies have shown higher tumor grades in mDLC [11,23,24]. However, a meta-analysis by Nasradani et al. showed a higher frequency of grade 1 in mDLC than in ILC [13]. An analysis found that while low and intermediate grades were associated with ILC when compared with mixed, tumor grade was not prognostic for ILC but was prognostic for mixed [14]. In our cohort, although mDLC tended to be higher grade, tumor grades were statistically similar between the two groups.

Some studies have found similar tumor diameters in mDLC and ILC, while others have observed smaller diameters in mDLC [12,13,14,23,24,25]. Additionally, lower rates of axillary metastasis and N positivity have been reported for mDLC [14,23,25]. However, it has also been claimed that axillary involvement is more common in mDLC [22,23]. In our study, the median tumor diameter was lower in mDLC, but the T stages showed similar characteristics. However, a more advanced N stage was observed in mDLC than in ILC.

Lymphovascular invasion (LVI), PNI and ECE are poor prognostic markers in breast cancer [27,28]. Some studies showed more LVI positivity in mDLC than in ILC [11,14]. Duraker et al., despite limited data availability, reported similar LVI and PNI rates [22]. Although lymph node metastasis in mDLC has been extensively studied, we did not find any study evaluating the ECE status. In our study, LVI positivity was detected at a higher rate in mDLC. However, PNI and ECE did not differ between the groups.

Contradictory findings exist regarding patterns of local recurrence and distant metastasis between the two groups [11,13,22,24]. Nazradini et al. reported no difference in metastasis sites between groups [13]. Ratha et al. noted more frequent bone metastases, locoregional recurrence, and distant metastasis in mDLC [11]. Other studies have observed similar rates of local and distant recurrence between groups [22,24]. In our cohort, although bone was the most common metastasis site and tended to increase in mDLC, the difference was statistically insignificant. There was no difference between the metastasis patterns of the patients. No difference was found in local recurrence rates, but the incidence of distant metastasis was statistically higher in mDLC. Zels et al. showed that biopsies from metastases in patients with metastatic mDLC have lobular features [15]. Although only two patients with mDLC were included, metastases that predominantly displayed lobular histology, independent of the predominant subtype of the primary tumor, were seen. This is consistent with publications that indicate that the clinicopathological features of mDLC are more closely related to those of ILC.

When examining survival data, Rakha et al. reported worse outcomes for mDLC than for ILC [11]. Conversely, Metzger-Filho et al. noted that in postmenopausal patients, mDLC showed better survival than that of ILC. Although a trend towards improvement was observed in multivariate modeling when comparing mDLC with ILC, the histological type was not defined as an effective factor in DFS and OS outcomes [14]. Similarly, various studies have shown that in multivariate analysis, the histological type has not been demonstrated as a prognostic factor for survival [22,23]. Xiao et al., in their extensive analysis of the Surveillance, Epidemiology, and End Results (SEER) database, performed a survival analysis based on histology, where the mDLC group showed better survival outcomes [26]. However, this study did not discuss the clinicopathological characteristics and treatment differences of the patients. The contradictory results for survival outcomes could be due to the retrospective analysis of the studies, limited cohort numbers, and inadequate balancing of the multifactorial influences that could affect the outcome. At the same time, regarding differences in the percentage of IDC in mDCL patients, genetically IDC-like or ILC-like characteristics of the tumor may cause contradictions in survival outcomes. In our study, no difference in OS and DFS was observed between the groups. We evaluated the confounding factors for OS and DFS as disease stage, age and treatments applied. However, since the confounding factors we mentioned did not differ between the groups, we thought that they had no effect on the survival difference between the groups. When the survival curves were analyzed, it was seen that after 12–13 years, the curves for both OS and DFS diverged between the groups to the detriment of mDLC. However, in multivariate modeling for OS, mDLC was identified as a poor prognostic factor. In our study, histopathology, PNI, tumor necrosis, and ECE were found to be statistically significant as prognostic factors affecting OS, and only ECE was found to be statistically significant as a prognostic factor affecting DFS in the multivariate analysis. When the clinicopathological features and treatments of the groups were analyzed, it was observed that the above prognostic factors and treatments did not differ between the groups. The survival results may not have reached statistical significance due to the similar distribution of prognostic factors between the groups.

Upon review of the existing studies in the literature, the clinical, pathological, and survival outcomes of both groups contain contradictions. There could be several reasons for these discrepancies. One reason could be related to the definition of mixed tumors. There was no official definition for mDLC until 2012. The Fourth Edition of the WHO classification of breast cancer defined mDLC as a tumor where a specific subtype makes up 50% of the tumor [6]. This definition was revised in the Fifth Edition to state that the specific subtype must constitute at least 10% of the tumor [1]. It remains unclear whether the current definition adequately describes the spectrum of pathology seen in these tumors and how this change might affect the outcomes of previous studies, including those conducted here. In general, the presence of a high-grade histological component in mixed tumors may be associated with aggressive clinical behavior. The increased presence of a high-grade histological component may further fuel aggressive behavior. Another potential reason for the discrepancies, especially in population-based studies, such as those using SEER data, could be that the International Classification of Diseases (ICD) codes do not reflect the actual pathology. Nasrazadani et al., in their study, compared ICD codes with pathology slides and noted that 50% of the tumors registered as mixed did not meet the WHO definition [12], complicating the evaluation of such studies. Zels et al. showed that biopsies from metastases in patients with metastatic mDLC have lobular features [15]. Although only two patients with mDLC were included, metastases that predominantly displayed a lobular histology, independent of the predominant subtype of the primary tumor, were seen. This is consistent with publications that indicate that the clinicopathological features of mDLC are more closely related to those of ILC. The effect of the percentage of different histologies in the tumor on the prognosis is controversial. It seems to be more important whether the tumor is genetically lobular-like or ductal-like. However, we do not have the possibility to perform these analyses.

It is necessary to mention the limitations of this study. Firstly, the retrospective design of the study and the potential selection biases that could arise should be considered. Although the number of patients might seem low, similar numbers are present in the literature due to the rarity of these tumors. Additionally, in this study, the percentage of existing components in mDLC tumors and the subtypes of lobular carcinoma were not specified.

## 5. Conclusions

Although the clinicopathological features of a new carcinoma may worsen when a ductal carcinoma accompanies lobular carcinoma pathologically, this result did not affect the survival outcomes in our study. Due to the conflicting data on the clinicopathological and survival outcomes of both groups, there is a need for larger prospective randomized trials with balanced confounding factors to clarify the clinical effects of mixed tumors.

Does the aggressive component determine survival in mixed tumors? What is the importance of the percentage of existing tumors? What are the factors that determine the component from which the metastasis will develop? Are the differences between the study results related to the fact that metastases may have developed from different components? It seems that more studies are needed to answer these questions.

## Figures and Tables

**Table 1:** Comparison of clinicopathologic characteristics.

	ILC N = 80 (61%)	mDLC N = 52 (39%)	*p* Value
Age (median, year)	54 (36–83)	52 (29–78)	**0.038**
Menopausal status			0.210
Pre-menopause	30 (37)	24 (46)	
Post-menopause	50 (63)	28 (54)	
Family history	25 (31)	16 (31)	0.555
Bilateral breast cancer	7 (9)	2 (4)	0.235
Stage			0.554
Stage I	11 (14)	6 (12)	
Stage II	30 (37)	15 (29)	
Stage III	34 (42)	25 (48)	
Stage IV	5 (6)	6 (11)	
T stage			0.127
T1	19 (24)	18 (35)	
T2	37 (46)	20 (39)	
T3	18 (23)	6 (11)	
T4	6 (7)	8 (15)	
N stage			**0.046**
N0	25 (31)	8 (15)	
N1	25 (31)	16 (31)	
N2	14 (18)	14 (27)	
N3	16 (20)	14 (27)	
Histological Subtypes			0.328
Luminal A	37 (46)	22 (42)	
Luminal B (HER-2 negative)	35 (44)	19 (36)	
Luminal B (HER-2 positive)	5 (6)	5 (10)	
HER-2 positive	1 (1)	4 (8)	
Triple Negative	2 (3)	2 (4)	
Hormon receptor status			
ER positive	76 (95)	41 (79)	**0.005**
PR positive	66 (83)	40 (77)	0.286
HER-2 positive	6 (8)	9 (17)	0.116
Grade (n = 132)			0.074
Grade 1	36 (45)	16 (31)	
Grade 2	34 (43)	25 (48)	
Grade 3	10 (12)	11 (21)	
Histopathological features			
Lymphovascular invasion (n = 107)	33 (52)	33 (77)	0.007
Perineural invasion (n = 106)	26 (40)	23 (56)	0.078
Intraductal component (n = 114)	48 (73)	37 (77)	0.381
Multi-centricity/focality (n = 121)	21 (29)	16 (33)	0.390
Tumor necrosis (n = 102)	9 (15)	12 (29)	0.079
Extracapsular invasion (n = 92)	29 (57)	30 (73)	0.080
Tumor diameter (median, cm)	2.3 (0.4–5.1)	2 (0.3–3.2)	**0.050**
Ki-67 (median, %)	10 (0–60)	20 (1–70)	0.064

ILC: Invasive Lobular Carcinoma, mDLC: Mixed Ductolobular Carcinoma, HER-2: Human epidermal growth factor receptor 2, ER: Estrogen receptor, PR: Progesterone receptor, Data are expressed as n (%) unless otherwise specified. Significant *p* value indicated in bold format.

**Table 2:** Comparison of treatment patterns.

	ILC N = 80 (61%)	mDLC N = 52 (39%)	*p* Value
Neoadjuvant chemotherapy			0.229
No	72 (91)	50 (90)	
Yes	7 (9)	2 (4)	
Surgery			0.761
No	3 (4)	1 (2)	
MRM	56 (70)	41 (79)	
BCS	21 (26)	10 (19)	
Axillary intervention			0.609
No	4 (5)	3 (6)	
SLNB	11 (14)	6 (11)	
AD	65 (81)	43 (83)	
Adjuvant chemotherapy			0.237
No	21 (26)	10 (19)	
Yes	59 (74)	42 (81)	
Adjuvant radiotherapy			0.304
No	23 (29)	12 (23)	
Yes	57 (71)	40 (77)	
Adjuvant hormonotherapy			0.273
No	11 (14)	10 (19)	
Yes	69 (86)	42 (81)	

ILC: Invasive Lobular Carcinoma, mDLC: Mixted Ductolobular Carcinoma, MRM: Modified Radical Mastectomy, BCS: Breast Conserving Surgery, SLNB: Sentinel Lymph Node Biopsy, AD: Axillary Dissection, Data are expressed as n (%) unless otherwise specified.

**Table 3:** Comparison of relapse and metastasis pattern and survival.

	ILC N = 80 (61%)	mDLC N = 52 (39%)	*p* Value
Local relapse			0.322
No	74 (9)	50 (96)	
Yes	6 (7)	2 (4)	
Metastasis			**0.029**
No	64 (80)	33 (64)	
Yes	16 (20)	19 (36)	
Bone metastasis			0.054
No	65 (81)	35 (67)	
Yes	15 (19)	17 (33)	
Brain metastasis			0.157
No	77 (96)	47 (90)	
Yes	3 (4)	5 (10)	
Lung metastasis			0.085
No	77 (96)	46 (89)	
Yes	3 (4)	6 (11)	
Liver metastasis			0.312
No	74 (93)	44 (89)	
Yes	6 (7)	6 (11)	
Overall survival			0.541
The 5-year (%)	80	76	
The 10-year (%)	64	67	
The 15-year (%)	59	52	
The median (month)	NR	186	
Disease-free survival			0.386
The 5-year (%)	73	68	
The 10-year (%)	59	62	
The 15-year (%)	59	42	
The median (month)	NR	165	

ILC: Invasive Lobular Carcinoma, mDLC: Mixed Ductolobular Carcinoma, NR: not reached, Data are expressed as n (%) unless otherwise specified. Significant *p* value indicated in bold format.

**Table 4:** The prognostic factors affecting overall survival in patients.

Overall Survival	Univariate Analysis			Multivariate Analysis		
	HR	95% CI	*p* Value	HR	95% CI	*p* Value
Histopathology						
ILC	1			1		
mDLC	1.2	0.66–2.17	0.543	2.95	1.10–7.88	**0.03**
Menopausal status						
Pre-menopause	1			1		
Post-menopause	2.16	1.14–4.11	**0.018**	3.16	0.80–12.40	0.099
Histological Subtypes						
Luminal A	1			1		
Luminal B (HER2-negative)	2	1.04–3.84	**0.037**	1	0.34–2.89	0.994
Luminal B (HER2-positive)	1.72	0.49–6.02	0.392	0.63	0.10–3.79	0.617
T stage						
T1	1			1		
T2	2.04	0.85–4.91	0.11	2.24	0.66–7.59	0.197
T3	2.16	0.78–5.99	0.137	1.71	0.42–6.83	0.448
T4	8.95	3.47–23.10	**<0.001**	2.51	0.67–9.40	0.17
N stage						
N0	1			1		
N1	2.31	0.62–8.57	0.208	1.15	0.20–6.34	0.87
N2	6.57	1.90–22.72	**0.003**	6.85	1.51–31.03	**0.012**
N3	9.91	2.90–33.84	**<0.001**	3.27	1.52–7.02	**0.003**
Grade						
Grade 1	1			1		
Grade 2	1.59	0.78–3.24	0.202	1.02	0.37–2.80	0.965
Grade 3	2.35	1.05–5.24	**0.036**	0.67	0.15–3.05	0.612
LVI						
Negative	1			1		
Positive	2.29	0.99–5.27	0.051	2.03	0.68–6.04	0.199
PNI						
Negative	1					
Positive	2.24	1.13–4.44	**0.02**	4.83	1.81–12.86	**0.002**
Tumor necrosis						
No	1			1		
Yes	2.49	1.24–5.01	**0.01**	1.7	0.66–4.35	0.266
ECE						
Negative	1			1		
Positive	2.92	1.27–6.75	**0.012**	5.07	1.41–18.22	**0.013**

ILC: Invasive Lobular Carcinoma, mDLC: Mixed Ductolobular Carcinoma, HR: Hazard Ratio, CI 95%: Confidence Interval, ECOG PS: Eastern Cooperative Oncology Group performance status, DM: Diabetes Mellitus, HER2: Human epidermal growth factor receptor 2, LVI: Lymphovascular invasion, PNI: Perineural Invasion, ECE: Extra capsular extension. Significant *p* value indicated in bold format.

**Table 5:** The prognostic factors affecting disease-free survival in patients.

Disease-Free Survival	Univariate Analysis			Multivariate Analysis		
	HR	95% CI	*p* Value	HR	95% CI	*p* Value
Histopathology						
ILC	1			1		
mDLC	1.27	0.73–2.21	0.391	0.63	0.27–1.45	0.281
Menopausal status						
Pre-menopause	1			1		
Post-menopause	1.71	0.95–3.08	0.07	1.02	0.37–2.76	0.966
T stage						
T1	1			1		
T2	2.01	0.88–4.58	0.095	1.18	0.32–4.34	0.796
T3	2.61	1.04–6.51	**0.039**	1.22	0.30–4.94	0.778
T4	8.16	3.30–20.19	**<0.001**	1.82	0.41–8.07	0.429
N stage						
N0	1			1		
N1	1.79	0.62–5.17	0.277	0.11	0.10–1.12	0.063
N2	4.58	1.67–12.51	**0.003**	0.62	0.07–5.09	0.658
N3	6.31	2.35–16.95	**<0.001**	1.06	0.13–8.19	0.954
Grade						
Grade 1	1			1		
Grade 2	1.65	0.86–3.19	0.131	0.75	0.27–2.09	0.585
Grade 3	2.17	1.00–4.69	**0.049**	0.88	0.20–3.73	0.865
PNI						
Negative	1			1		
Positive	2.19	1.15–4.18	**0.017**	2.2	0.92–5.24	0.073
Tumor necrosis						
No	1			1		
Yes	2.28	1.15–4.52	**0.018**	0.99	0.39–2.53	0.995
ECE						
Negative	1			1		
Positive	2.18	1.03–4.59	**0.04**	3.157	1.138–8.763	**0.027**

ILC: Invasive Lobular Carcinoma, mDLC: Mixed Ductolobular Carcinoma, HR: Hazard Ratio, CI 95%: Confidence Interval, PNI: Perineural Invasion, ECE: Extra capsular extension. Significant *p* value indicated in bold format.

## Data Availability

The datasets used and/or analyzed during the current study are available from the corresponding author upon reasonable request.

## Abbreviations

BC	breast cancer
mDLC	mixed duktolobuler Carcinoma
İLC	invaziv lobuler carcinoma
IDC	invaziv ductal carcinoma
ER	estrogen receptor
PR	progesterone receptor
HER2	human epidermal growth factor receptor 2
AJCC	the American Joint Committee on Cancer
BRCA1	breast cancer1
FHIT	fragile histidine triad protein
ECE	exstracapsuler extension
PNI	perineural invasion
LVI	lenfovascular invasion
WHO	World Health Organization
SEER	Surveillance, Epidemiology, and End Results
FISH	fluorescence in situ hybridization
OS	overall survival
DFS	disease free survival
